# Quality indicators for occupational therapy: a scoping review

**DOI:** 10.1186/s12913-024-11548-1

**Published:** 2024-09-12

**Authors:** Thomas Ballmer, Sara Frey, Andrea Petrig, Brigitte E. Gantschnig

**Affiliations:** 1 Institute of Occupational Therapy, School of Health Sciences, ZHAW Zurich University of Applied Sciences, Winterthur, Switzerland; 2 Swiss National Association of Occupational Therapy (EVS/ASE), Bern, Switzerland; 3 Department of Rheumatology and Immunology, University Hospital (Inselspital) and University of Bern, Bern, Switzerland

**Keywords:** Occupational therapy, Evaluation, Reimbursement, Quality improvement, Quality indicators

## Abstract

**Background:**

Occupational therapists are increasingly asked to demonstrate the effectiveness, appropriateness, and efficiency of their interventions to funding bodies. However, the extent to which this is practiced and the health policy context within which such a practice is situated differs internationally. The aim of this scoping review was to establish which quality indicators are used internationally for this purpose.

**Methods:**

We conducted a scoping review, limiting our search to Europe and the English-speaking world. To search for suitable literature, we used specialized databases from medicine, health sciences, and related fields, including CINAHL Complete and MEDLINE, as well as free internet search via Google. Furthermore, we contacted national occupational therapy associations from several countries asking for access to documents found within this search that were only accessible to association members.

**Results:**

The screening process identified 32 studies and documents from six national contexts. We identified and described process-level indicators, functional outcome indicators, one outcome indicator based on individual goal attainment (the Goal Attainment Scale, or GAS), and PRO-Ergo, a patient-reported experience measure (PREM). There was little information on the use of quality indicators to demonstrate the effectiveness, appropriateness, and efficiency of occupational therapy services to funding bodies in Europe and the English-speaking world that was openly available. The identified process indicators were in most cases not specific to occupational therapy interventions. Functional outcome indicators were highly specific to certain client groups or health conditions and partially appropriate for use in occupational therapy. The GAS was found to be a highly customizable measure which allowed an evaluation on body structure and function levels as well as activity and participation levels. PRO-Ergo was focused on the clients’ subjective view and their experience with occupational therapy interventions.

**Conclusions:**

All identified quality indicators have advantages and disadvantages. Process-level indicators specific to occupational therapy could be a chance to foster the use of best practice methods. GAS and PRO-Ergo seem to be the most versatile assessment, allowing an evaluation on the level of activity and participation. Functional outcome indicators that cover a broad area of client problems may be useful additional quality indicators for some areas of practice.

## Background

Against a background of rising health care costs and increasing demands for health care services, the need to improve efficiency, appropriateness, and effectiveness of health care service provision is becoming more pressing [1]. In these efforts, quality indicators play a key role. Quality indicators are measures that allow for the quantification of the quality of different aspects of the structures, processes, and outcomes of health care provision [2–4]. Structural indicators operate on a health system or organizational level. They commonly refer to the use and/or accessibility of resources, for instance the rate of patients per doctor or access to specialised health technologies [4]. Process-level indicators represent how and what kind of services have been provided to patients, for instance the “Proportion of patients assessed by a doctor within 24 hours of referral” or the “Proportion of patients treated according to clinical guidelines” [4]. Outcome indicators refer to the results of these services, for instance the change in functional status for patients with knee impairments [5].

These indicators can be reported in the form of rates or proportions (e.g., percentage of patients 65 years old or older discharged home within 4 weeks following hospital treatment for hip fracture) or of means or averages (e.g., patients’ mean improvement on a test of mobility from admission to discharge) [4, 6]. The interpretation of these measures allows stakeholders within health systems to identify areas of service provision that excel or lack in terms of efficiency, appropriateness, and effectiveness. This information can serve as a basis for health systems-related decision-making [6], be it the direct allocation of resources or the creation of incentives for improvement, sometimes by tying reimbursement for various actors (e.g., hospitals, doctors/ therapists in private practice) to their performance on quality indicators [2]. Thus, the way that these quality indicators are designed has far reaching consequences for health policy, but also the day-to-day reality of health care professionals, as well as the health and well-being of their patients [2].

Occupational therapists are no exception when it comes to this increasing demand to demonstrate the effectiveness, appropriateness, and efficiency of health care services. Switzerland is one of the countries where this demand is currently being codified into law more explicitly. The Swiss Federal Act on Health Insurance (HIA; Art. 58 et seq.) and the Ordinance on Compulsory Health Care (OAMal; Art. 77, para 1), revised in 2021, obliges service providers and health insurers to enter into nationwide contractual agreements on quality development. These quality contracts regulate, among other things, the measurement of quality, and therefore require the definition of suitable quality indicators.

However, the question what quality indicators are ‘suitable’ is not always easily answered. Quality indicators are often highly specific to a certain health care setting or patient group and cannot be easily adapted to other areas or groups [2], which in turn creates the need for a large amount of different indicators. Furthermore, it has been criticized that not all quality indicators are appropriate to represent the contributions of different health professions to health outcomes on different levels specific to those professions (e.g., body function vs. activity/ participation levels) [7]. It stands to reason that quality indicators used to demonstrate quality of service provision to funding bodies need to be able to reflect plausible results of the intervention that is being funded.

This scoping review was commissioned by the Swiss National Association of Occupational Therapy (EVS/ASE) to serve as a basis for the definition of quality indicators in contractual quality agreements with the two large health insurance associations in Switzerland, focusing on process-level and outcome indicators. For the aforementioned reasons, the results of this review are, however, of interests not only to occupational therapists and their partners in the health care system, but to health professionals in general.

## Method

The aim of this scoping review was to establish an overview of quality indicators that are being used internationally (focusing on Europe and the English-speaking world) to demonstrate the effectiveness, appropriateness, and/or efficiency of occupational therapy interventions to funding bodies and, if applicable, whether experience exists regarding the suitability of these quality indicators. In the following, we will describe the framework and method we used in this process.

### World Federation of Occupational Therapists quality indicators framework

In compiling an overview of currently known quality indicators for occupational therapy, we used the World Federation of Occupational Therapists’ (WFOT) *Quality Indicators Framework* matrix as a guide for the identification of indicators [3]. The framework was created in response to the increasing demand on occupational therapists to demonstrate the effectiveness, appropriateness, and/or efficiency of their interventions, and to foster the development and use of quality indicators appropriate to occupational therapy practice. The framework conceptualizes different types of quality indicators for occupational therapy using a matrix whose vertical axis consists of quality dimensions, while the horizontal axis represents quality perspectives (see Table 1). In the *Structure* column, the availability of the appropriate number of competent occupational therapists in the right place at the right time is addressed, as well as the question of “whether other types of physical, financial, technical, and social resources necessary to provide quality occupational therapy services are continuously available in an economic, socially and environmentally sustainable manner” [3]. As the column name implies, these questions are situated on a structural level and regard questions of health care policy and workforce planning. In the *Process* column, “the ability of intended users to access occupational therapy” [3] also seems situated more in that era, while the categories *optimal use of resources* as well as *success in attaining occupational therapy goals* in the *Outcome* column directly refer to the effectiveness and efficiency of occupational therapy interventions. Lastly, *Satisfaction throughout service delivery* addresses the client perspective, while *Incidents resulting in harm* addresses patient safety and critical incidents.

**Table 1:** World Federation of Occupational Therapy quality indicator framework

	Structure	Process	Outcome
**Appropriateness**: Right service, person, place, time	Availability of competent occupational therapists.		
**Sustainability**: Access to resources without compromising future availability	Long term supply of resources.		
**Accessibility**: Ease in obtaining services		Ability to access service	
**Efficiency**: Use of resources for maximum results		Optimal use of resources.	
**Effectiveness**: Evidence-informed services for those who benefit			Success in attaining occupational therapy goals.
**Person-Centeredness**: Experiences of receiving service			Satisfaction throughout service delivery.
**Safety**: Reduction of risk and avoidance of harm			Incidents resulting in harm

Based on the guide to the WFOT QUEST Quality Evaluation Strategy Tool [3]

For the purpose of this review, we focused on the columns *Process* and *Outcome* and the rows *Efficiency*,* Effectiveness*, and *Person-Centeredness.* The column *Structure*, as laid out above, is situated more on a health and educational policy level, while the row *Safety* was outside the purview of our study because relevant indicators had already been defined by the contractual partners (see above).

### Scoping review

To answer the question which quality indicators are used internationally to demonstrate the effectiveness, appropriateness, and efficiency of occupational therapy interventions to funding bodies, we conducted a literature review. Since we assumed the available literature on the topic to not be primarily comprised of scientific studies, but to also include other documents of diverse provenance (e.g., strategy documents, magazine articles), we chose the *scoping review* method. Scoping reviews allow for greater flexibility in terms of the types of texts included compared to other types of literature reviews [8].

### Data collection

We conducted this scoping review in July 2023 based on the Joanna Briggs Institute manual on evidence synthesis [9]. After formulating the research question, we defined initial relevant keywords (see Table 2), inclusion and exclusion categories. To search for suitable literature, we used specialized databases from medicine, health sciences, and related fields, including CINAHL Complete and MEDLINE, as well as free internet search via Google. Furthermore, in collaboration with the Swiss Association of Occupational Therapy (EVS/ASE), we contacted several national occupational therapy associations, as well as other international contacts of the EVS/ASE, to ask for access to documents found within this search that were only accessible to association members.

**Table 2:** Search terms used in the document search

“Quality indicators”			“Occupational therapy”			
Keywords		Subject Headings	Keywords			Subject Headings
English	German		English	German		
quality indicator* quality assurance quality management quality measure* clinical indicator* efficacy efficiency impact evidence indication index	Qualitätsindikator* Qualitätssicherung Qualitätsmanagement Wirksamkeit Effektivität Zweckmässigkeit Evidenz	Quality Indicators Quality Assurance Guideline Adherence Quality of Health Care Quality Assessment Health Status Indicators Clinical Indicators Quality of Care Research	occupational therap*	Ergotherap*		Occupational Therapy Research, Occupational Therapy Occupational Therapy Practice Occupational Therapy Service Occupational Therapy Practice, Research-Based Occupational Therapy Practice, Evidence-Based Occupational Therapy Assessment
“Reimbursement”			“Funding bodies”			
Keywords		Subject Headings	Keyword			Subject Heading
English	German		English	German		
reimbursement remuneration payment	Vergütung Finanzierung	Reimbursement, Incentive Insurance, Health, Reimbursement	funding* insurance	Kostenträger Versicherung* Krankenkasse* Pflegekasse*		Insurance Insurance, Health, Reimbursement Government Agency

### Data analysis

We screened the documents we found using the online platform Covidence [10]. The selection process was carried out by the first and second author. In a first step, we screened titles and abstracts of documents and included or excluded the documents based on defined criteria (see Table 3). To resolve conflicts between the two reviewers regarding inclusion or exclusion, we consulted another team member, the last author. In the following step, we applied the same procedure for the included full texts. This time, the two reviewers discussed any disagreements and decided if the document would be included or excluded. Then, we extracted data relevant to the research question from the documents and synthesized the data.

**Table 3:** Inclusion and exclusion criteria

Inclusion criteria	Exclusion criteria
The study or document concerns quality indicators (e.g., assessments, measures, etc.) that are used to demonstrate the effectiveness, appropriateness, and/or efficiency of occupational therapy towards funding bodies (e.g., insurance companies).	The study or document is older than 2000.
The study or document is in German or English (machine translatable documents in other languages can be included).	The study or document concerns other types of quality assurance (e.g., internal to organizations or associations) or indicators used for other purposes (e.g., national registries).
The study or document refers to Europe, North America, Australia and/or New Zealand	

## Results

**Fig. 1:**
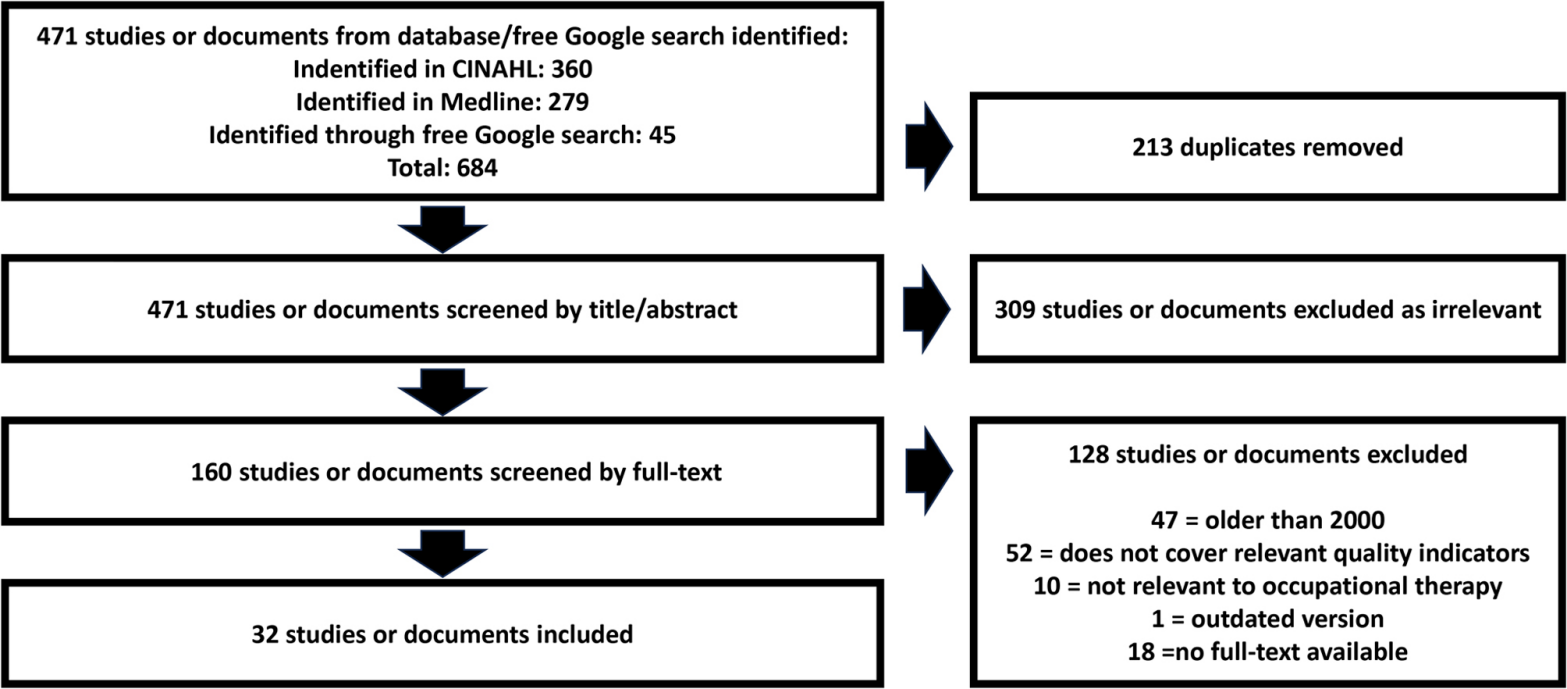
Screening process studies and documents

The screening process identified 32 studies and documents from six national contexts. In Fig. 1, the screening process is visualized. In Table 4, the number of documents and studies per country and a short description of the relevant quality indicators (if any) identified is provided. Although we used due diligence in our search process, it is highly likely that some relevant documents were neither openly available on the internet nor the subject of articles in specialized databases.

**Table 4:** Quality indicators that have been identified in the scoping review

Quality indicators identified	Country	Documents	Additional communication (e.g., with representatives of professional associations)
Merit-Based Incentive Payment System (MIPS) Quality Indicator Sets Section GG Self-Care (Activities of Daily Living) and Mobility Items form	USA	18	no
Goal Attainment Scale (GAS)	Switzerland	3	no
NICE Indicator List	UK	1	no
Quality Improvement Plan for Ontario Health Teams (QIPOH) Indicator List	Canada	1	yes (collaborators in development of WFOT quality indicators framework)
None	Germany	3	yes (representatives of professional association)
PRO-Ergo	Netherlands	1	yes (additional international contact)
None	International or multiple countries	5	no
	**Total**	**32**	

### United States of America

Most documents identified in our review were situated in the US-American context and were concerned with the provision of quality indicators to demonstrate effectiveness, appropriateness, and/or efficiency of health care services to Medicare. Medicare is a public health insurance program that provides health care coverage for Americans 65 years old or older and certain younger people with disabilities [11]. Medicare consists of three parts that cover hospital insurance (part A), medical insurance (part B), and prescription drug coverage (part C). In the available documents, mainly two reimbursement systems were described: the *Merit-Based Payment System* (MIPS) and the *Outcome and Assessment Information Set* (OASIS), which will be the focus of our analysis. Other similar systems, namely the Minimum Data Set and the CARE-tool used in hospital settings, were also mentioned [12–15]. Private insurers have their own criteria for reimbursement, but they are laid out less transparently than in the case of Medicare [16, 17].

### Merit-based incentive payment system (MIPS)

The Merit-Based Incentive Payment System (MIPS) is the main reimbursement system currently in use for reimbursing health care services provided to patients covered under Medicare. MIPS is concerned with the reimbursement of part B services. It is part of the Quality Payment Program (QPP) that is based on the Medicare Access and CHIP Reauthorization Act (MACRA) of 2015. The aim of establishing QPP was to base reimbursement more on quality of care [18]. Eligible health care professions include physicians, nurse practitioners, occupational therapists, physical therapists, and several other professions.

Under MIPS, clinicians yearly report data in four areas: *quality*,* improvement activities*,* promoting interoperability*, and *cost*. *Cost* is calculated automatically based on claims submitted to Medicare. In *promoting interoperability*, clinicians report on a set of measures and objectives connected to digitization (e.g., use of electronic health records, e-prescribing). In the area *improvement activities*, clinicians have to attest to between 2 and 4 predefined activities that improve access to care, enhance client engagement, and/or improve processes. Finally, in the area *quality*, clinicians are asked to provide at least six quality measures, one of which must be an outcome measure or another high priority measure. These measures must be provided for a minimum of 70% of patients over the respective 12-month period [19]. *Centers for Medicare & Medicaid Services* (CMS), the federal agency responsible for Medicare and Medicaid, has defined measures suitable for physical and occupational therapists (see Table 5). However, it has been proposed that quality indicators that are less generic and more reflective of the contribution of occupational therapists be developed and included [20].

**Table 5:** 2023 MIPS quality measures for physical therapy/ occupational therapy

Measure title	Measure Nr.	Measure type
Urinary Incontinence: Assessment of Presence or Absence of Urinary Incontinence in Women Aged 65 Years and Older	048	Process
Urinary Incontinence: Plan of Care for Urinary Incontinence in Women Aged 65 Years and Older	050	Process – High Priority
Diabetes Mellitus: Diabetic Foot and Ankle Care, Peripheral Neuropathy – Neurological Evaluation	126	Process
Diabetes Mellitus: Diabetic Foot and Ankle Care, Ulcer Prevention – Evaluation of Footwear	127	Process
Preventive Care and Screening: Body Mass Index (BMI) Screening and Follow-Up Plan	128	Process
Documentation of Current Medications in the Medical Record	130	Process – High Priority
Preventive Care and Screening: Screening for Depression and Follow-Up Plan	134	Process
Falls: Plan of Care	155	Process – High Priority
Rheumatoid Arthritis (RA): Functional Status Assessment	178	Process
Elder Maltreatment Screen and Follow-Up Plan	181	Process – High Priority
Functional Outcome Assessment	182	Process – High Priority
Functional Status Change for Patients with Knee Impairments	217	Patient-Reported Outcome-Based Performance Measure – High Priority
Functional Status Change for Patients with Hip Impairments	218	Patient-Reported Outcome-Based Performance Measure – High Priority
Functional Status Change for Patients with Lower Leg, Foot or Ankle Impairments	219	Patient-Reported Outcome-Based Performance Measure – High Priority
Functional Status Change for Patients with Low Back Impairments	220	Patient-Reported Outcome-Based Performance Measure – High Priority
Functional Status Change for Patients with Shoulder Impairments	221	Patient-Reported Outcome-Based Performance Measure – High Priority
Functional Status Change for Patients with Elbow, Wrist or Hand Impairments	222	Patient-Reported Outcome-Based Performance Measure – High Priority
Preventive Care and Screening: Tobacco Use: Screening and Cessation Intervention	226	Process
Dementia: Cognitive Assessment	281	[not described]
Dementia Associated Behavioral and Psychiatric Symptoms Screening and Management	283	Process
Dementia: Safety Concern Screening and Follow-Up for Patients with Dementia	286	Process – High Priority
Dementia: Education and Support of Caregivers for Patients with Dementia	288	Process – High Priority
Falls: Screening for Future Fall Risk	318	[not described]
Functional Status Change for Patients with Neck Impairments	478	Patient-Reported Outcome-Based Performance Measure – High Priority
Screening for Social Drivers of Health	487	Process – High Priority

Adapted from Centers for Medicare and Medicaid Services [5]

Each MIPS area is scored individually. For instance, the *quality* area is scored based on the completeness of the required data and their quality in relationship to benchmarks. These benchmarks are based on performance data from a baseline period (usually two years prior to the reporting year). The area scores are weighted and transformed into a total MIPS score between 0 and 100 points. Clinicians that score below 75 points will suffer a negative payment adjustment through Medicare of up to -9%. Clinicians that score 75 points and above will receive a positive payment adjustment. The factor depends on statutory budget neutrality requirements (i.e., how much money is available under an existing budget). If a clinician scores 89% or above, they will receive an additional payment adjustment for exceptional performance, again depending on budget neutrality requirements [19]. As of 2020, occupational therapists only needed to report in the MIPS areas *quality* and *performance improvement.* These areas were, therefore, reweighted so that quality accounted for 85% and performance improvement for 15% of the total MIPS score [21, 22].

The MIPS requires a lot of reporting and has been described as at times “tedious” [18]. In 2021, 3.31% of clinicians suffered a negative payment adjustment, while 86.12% achieved a positive payment adjustment, with 77.86% achieving an additional adjustment for exceptional performance. As the number of eligible clinicians changed drastically between 2020 and 2021 due to changes in eligibility rules, it is difficult to compare 2021 data with earlier years [23]. The Covid-19-pandemic is another factor that makes it harder to draw conclusions on the performance of the new system. Furthermore, MIPS has been criticized by the American Occupational Therapy Association for being physician-centred and not sufficiently reflective of the services of non-physician health professionals [7].

### Section GG self-care (activities of daily living) and mobility items

The *Section GG Self-Care (Activities of Daily Living) and Mobility Items* form [24] is a form used over different settings (skilled nursing facilities, home health care, inpatient rehabilitation) to evaluate self-care skills and activities of daily living (see Table 6). While it is not an explicit occupational therapy assessment - the *Centers for Medicare and Medicaid Services* solely state that it should be coded by *qualified clinicians* [25] – it is often used by occupational therapists, and occupational therapists have been urged to demonstrate their contribution to the interprofessional team by claiming this task [20, 21].

**Table 6:** Items included in the section GG self-care (activities of daily living) and mobility items form

Admission	Goal	Discharge	Item
-	-	-	Eating
-	-	-	Oral Hygiene
*-*	*-*	*-*	*Toilet hygiene*
*-*	*-*	*-*	*Wash upper body*
*-*	*-*	*-*	*Shower/bathe self*
*-*	*-*	*-*	*Upper body dressing*
*-*	*-*	*-*	*Lower body dressing*
*-*	*-*	*-*	*Putting in/taking off footwear*

The form is rated at admission and discharge on a 6 to 1 scale with 6 = Independent; 5 = Setup; 4 = Supervision/ Touching; 3 = Partial Assistance; 2 = Substantial Assistance; 1 = Dependent; additionally, the following codes are used: 07 = Refused; 09 = Not Applicable; 10 = Not attempted due to environment limitations; 88 = Not attempted due to medical condition/safety. Adapted from the American Occupational Therapy Association [24]

### Switzerland

**Fig. 2:**
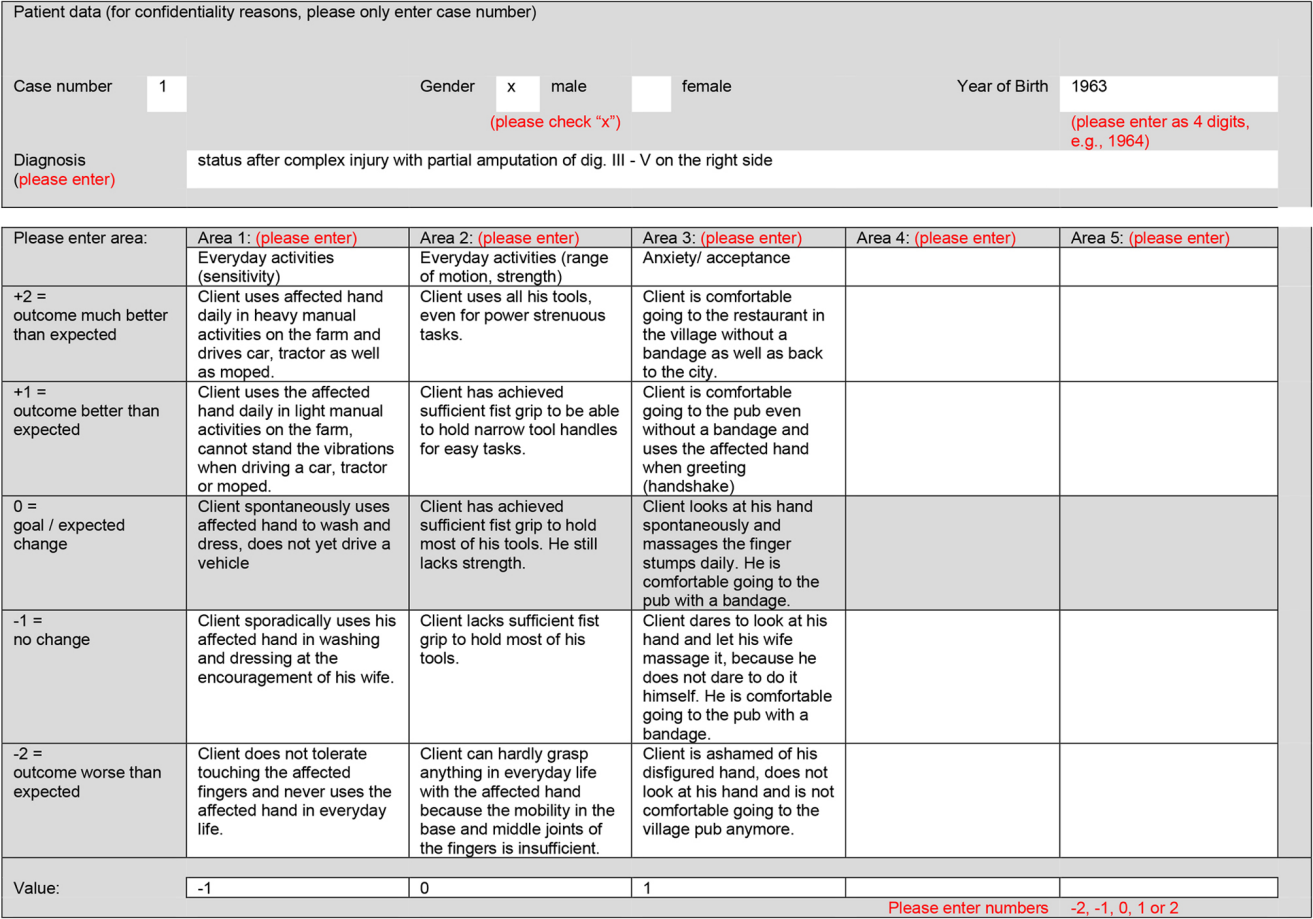
Example of a completed Goal Attainment Scale. Note: adapted from EVS/ASE [28]

In Switzerland, there has been a contractual quality agreement between the Swiss Association of Occupational Therapy (EVS/ASE) and the associations of private insurances dating back to 2011 [26]. Since 2019, this agreement has been expanded to include not only general health insurance, but also accident, disability, and military insurance cases. The effectiveness of occupational therapy services is evaluated using the Goal Attainment Scale (GAS) as a quality indicator. GAS [27] is a standardized, valid, and reliable assessment that expresses the degree of achievement of individually set goals in a numerical value from − 2 (worse outcome than expected) to + 2 (much better outcome than expected) (see Fig. 2).

All self-employed occupational therapists as well as occupational therapy organizations and their employees are obliged to record five cases with the GAS and document them on an online platform each year. 10 cases per language region are randomly selected and checked for content quality. Reasons for non-participation or incomplete participation must be declared on the online platform. Unjustified non-participation can be sanctioned [29]. The implementation of this procedure was accompanied by a research project evaluating the quality and content of goal setting by Swiss occupational therapists [30].

In 2020, 2159 occupational therapists were registered on the online platform, documenting 8106 clients [29]. The number of registered therapists has continuously risen from 2016, when there were 1265, to 2020. Reported outcomes in terms of goal attainment have remained stable in this time frame. Goals seem to have been set in a realistic, measurable manner. These results have been deemed as positive by representatives of all contractual parties [29].

### The Netherlands

#### PRO-Ergo questionnaire (patient reported experience measure)

While information on the use of quality indicators for reimbursement by Dutch occupational therapist was not to be found online using our search terms, representatives of the Dutch professional association that were contacted directly by the EVS/ASE reported that they are not asked to provide quality indicators to funding bodies (EVS/ASE, personal communication, October 2023). However, it was also reported to us through personal contacts (J. Leenders, personal communication, September 2023) that some Dutch insurance companies do require occupational therapists to provide the PRO-Ergo questionnaire [31], a patient reported experience measure (PREM) that includes a number of statements on activities, self-management, social environment, and satisfaction with occupational therapy services that are rated on a scale of 0 to 10 (see Table 7).

**Table 7:** Questions included in PRO-Ergo used by some Dutch occupational therapists

1. I can carry out my daily activities as I want.
2. I am satisfied with performing my daily activities indoors, with or without with help/ aids (e.g., washing, dressing, cooking, cleaning, hobbies).
3. I am satisfied with my participation in activities outside the home, with or without help/ aids (e.g., shopping, outings, work, school, appointments).
4. I have insight into the possibilities and limitations resulting from my condition/ disease.
5. I ask for help, when I need it (e.g., in doing everyday things).
6. I can indicate my limits.
7. I am satisfied with the way I distribute my energy so that I can carry out my daily activities.
8. I accept the consequences of my condition/ disease.
9. I can (practically) cope with the consequences of my condition/ disease.
10. My environment (partner/neighbours) accepts the consequences of my condition/ disease.
11. My environment (partner/next-of-kin) can (practically) cope with the consequences of my condition/ disease.
12. Because of occupational therapy, I can do my daily activities better.
13. I would recommend others with similar symptoms to get occupational therapy.

Adapted from Ergotherapie Nederland [31]; translated by the authors

Unfortunately, we could not identify any descriptions of experiences with this measure.

Apart from this, occupational therapists’ role in reimbursement for therapeutic aids is described in the literature, including standardised measures for funding bodies. However, this does not concern the reimbursement of occupational therapy services themselves [32].

### Other countries

Apart from USA, Switzerland, and the Netherlands, little information was available on quality indicators used by occupational therapists to demonstrate the effectiveness, appropriateness, and/or efficiency of their services to funding bodies. For the UK and some Canadian provinces, we found the use of more general quality indicators that are non-specific to occupational therapy and mainly process-level, e.g., “The percentage of patients with hypertension aged 16 to 74 years in whom there is an annual assessment of physical activity, using GPPAQ, in the preceding 15 months” and similar [33, 34]. In Germany, occupational therapists are not providing quality indicators to funding bodies in the outpatient sector, while the indicators used in the inpatient sector are limited to the amount and/ or duration of occupational therapy sessions (Deutscher Verband Ergotherapie, personal communication, June 2023). However, the development of quality indicators is a stated goal of the German professional association of occupational therapists (DVE), as stated in a current position paper [35, 36], and has been for several years [37]. Representatives of the Swedish professional association that were contacted directly by the EVS/ASE reported that they are not asked to provide quality indicators to funding bodies (EVS/ASE, personal communication, October 2023).

## Discussion

The aim of this scoping review was to establish which quality indicators are used nationally and internationally (focusing on Europe and English-speaking countries abroad) to demonstrate the effectiveness, appropriateness, and/or efficiency of occupational therapy interventions to funding bodies and, if applicable, whether experience exists regarding the suitability of these quality indicators. There was little information that was openly available, which could mean that quality indicators are either not in widespread use, that information on their use is not accessible, or both. While we can, therefore, not claim to give a complete overview on all quality indicators used for occupational therapy services in Europe and English-speaking countries abroad, the reporting systems that we have identified in our opinion do show a certain breadth of the possible use of quality indicators in this field. In essence, we identified two reporting systems that utilise process-level indicators, two that utilise outcome-level indicators, and one the utilises both types of indicators. Table 8 visualizes how these systems can be organized within the WFOT Quality Indicator Framework.

**Table 8:** Identified quality indicators organized using the WFOT Quality Indicator Framework

	Structure	Process	Outcome
**Appropriateness**: Right service, person, place, time			
**Sustainability**: Access to resources without compromising future availability			
**Accessibility**: Ease in obtaining services			
**Efficiency**: Use of resources for maximum results		NICE indicator list (UK) QIPOH indicator list (CAN) MIPS process indicators (USA)	
**Effectiveness**: Evidence-informed services for those who benefit			Goal Attainment Scale (CH) MIPS outcome indicators (USA) Section GG Self-Care and Mobility Items (USA)
**Person-Centerdness**: Experiences of receiving service			Patient Reported Experience Measure (NL)
**Safety**: Reduction of risk and avoidance of harm			

Based on the guide to the WFOT QUEST Quality Evaluation Strategy Tool [3]

### Process-level indicators

The process-level indicators identified as part of the National Institute for Health and Care Excellence (NICE) menu of indicators [33] and the Quality Improvement Plan for Ontario Health Teams (QIPOH) indicator list [34] mainly concern the percentage of patients over a certain reporting period for whom a certain intervention, procedure, test, or similar has been carried out. The process-level indicators on the *2023 MIPS Quality Measures List* [5] for physical therapy and occupational therapy serve the same purpose, but on the individual client level (i.e., has a certain intervention, procedure, test or similar been carried out for this client). This allows the responsible agencies or funding bodies to assess the degree to which the reporting professionals or institutions are adhering to best practice guidelines or similar, either in general (NICE, QIPOH) or on an individual level (MIPS).

However, the NICE menu of indicators and the QIPOH indicator list include few, if any, quality indicators that are appropriate to reflect occupational therapy interventions. Some of the process-level indicators in the MIPS Quality Measures List are more appropriate to reflect the specific contributions of occupational therapy to health outcomes (e.g., related to fall risk management or functional status assessment). However, they are also very specific to certain client groups and/ or health conditions (e.g., “Rheumatoid Arthritis (RA): Functional Status Assessment”).

### Outcome indicators

Unlike process-level indicators, outcome indicators necessarily operate on an individual level. The outcome indicators on the *2023 MIPS Quality Measures List* [5] for physical- and/or occupational therapy are very well suited to record the outcomes of occupational therapy interventions, but also very specific to certain client groups and/or health conditions (e.g., “Functional Status Change for Patients with Elbow, Wrist or Hand Impairments”). The Section GG Self-Care (Activities of Daily Living) and Mobility Items form, on the other hand, is not specific to certain client groups and/ or health conditions, but appropriate for all clients that have problems in performing activities of daily living (ADL) – which is a key domain for occupational therapy [38]. In content and scoring, Section GG is very similar to ADL assessment forms like the Barthel index [39] that, while not necessarily specific to occupational therapy, are also commonly used by occupational therapists.

The GAS [27] works on a different level than the outcome indicators included in the MIPS and the Section GG form as it does not measure objective functional change, but *goal attainment*. As demonstrated in Fig. 2, this does not only make it possible to assess functional change on the level of activity and participation (e.g., “Client has achieved sufficient fist grip to be able to hold narrow tool handles for easy tasks”) beyond ADLs, but also to assess facets of change relevant to clients that are not considered by strictly functional assessments, like psychosocial aspects (e.g., “Client is comfortable going to the pub even without a bandage and uses the affected hand when greeting (handshake)”). It is also not specific to a certain client group or a specific health condition but can be used as a generic assessment across all domains of occupational therapy (and, potentially, other professions).

While the GAS relies on specific, measurable goals [27], PRO-Ergo is, as a PREM, by definition a subjective assessment. It assesses not only the subjective outcome of an intervention, but also the client’s satisfaction with that intervention. Where GAS is a client- or patient-centred assessment in the collaborative setting of goals, PRO-Ergo is fully focused on the client’s subjective experience of the intervention and its outcome.

To synthesize these findings, all indicators we have identified in the literature have advantages and disadvantages. Process-level indicators allow the responsible agencies or funding bodies to assess the degree to which the reporting professionals or institutions are adhering to best practice. However, using these with occupational therapists would necessitate the creation of specific process-level indicators for this purpose, based on guidelines for best practice.

Specific functional outcomes like the ones included in the MIPS Quality Indicator List [5] are an appropriate way to demonstrate functional change as an outcome of an occupational therapy intervention for persons with acute and/or chronic health conditions. They are, however, often highly specific to certain client groups and health conditions. To utilize this kind of functional outcomes for quality indication for occupational therapy across the board, there would need to be a large pool of items to draw from to cover the breadth of occupational therapy practice. In contrast, Section GG [25], an assessment used for the evaluation of ADL skills, is more general. It or a similar assessment could possibly be used across all fields that require an assessment of ADL.

The GAS [27] and PRO-Ergo [31] have the advantage that they are usable across all fields of practice and client groups, provided that certain clients are not able to participate in the assessment process (e.g., persons with severe dementia), necessitating the involvement of proxies (e.g., significant others). Both, make it possible to assess outcomes on the activity and participation level. Between the two assessments, GAS is the more labour-intensive, as individual collaborative goals are defined in collaboration with the clients, which reflects the professions client-centred approach.

Given that all different kinds of quality indicators have their advantages and disadvantages, the implementation of a specific kind of quality indicator for a specific health profession will likely be shaped by current health policy priorities on one side and the interest of practitioners, represented by professional associations and/ or unions, on the other side. In Switzerland, these interests and priorities dovetail for occupational therapy when it comes to using quality indicators to maintain or increase standards of care [40, 41]. Because another urgent goal of Swiss health policy is containing health care spending [40], it is important that representatives of health care professionals’ interests (i.e. professional associations, unions) take an active role in shaping the implementation of quality indicators. The use of quality indicators can also be an opportunity for smaller health care professions to sharpen their profile and demonstrate their value to funding bodies and policymakers. However, to achieve the latter, quality indicators have to be able to represent the specific contribution of this profession to clients’ health and well-being (e.g., in the case of occupational therapy, gains in independence, autonomy and/ or participation).

On the other hand, complex quality indicator reporting systems have been criticized for placing a high administrative burden on practitioners (Khullar et al. 2021). Beyond that, health care policy makers may try to increase efficiency by tying health care provider reimbursement to their performance on quality indicators (which is already the case in some areas of the MIPS). This can however have the unwanted effect of providers prioritizing care that is easily measurable instead of what is best for the individual client (Wagenschieber & Blunck, 2024). A focus on more flexible and client-centred outcome indicators could possibly help mitigate these risks.

While we have been focussing on occupational therapy, most of the quality indicator systems we have looked at (e.g., MIPS, NICE, QIPOH, GAS) in this study are used by multiple health care professions. Challenges and opportunities are similar, and we therefore believe that these results are transferable to other professions.

### Limitations

While we have identified little information on quality indicators used to demonstrate effectiveness, appropriateness, and efficiency of occupational therapy services to funding bodies, we cannot say for certain if this is due to their limited use or to the limited accessibility of relevant information. We were also not able within this study to examine quality indicators being used beyond Europe and the English-speaking world. Also, we were not able to elicit much information on stakeholders’ experiences with these indicators and their usefulness. This is mainly due to the apparent dearth of studies that explore these experiences.

### Further research

While results of this study have added additional dimensions to quality indicator frameworks like the WFOT QUEST [3], further research into stakeholders’ (e.g., occupational therapists, insurance companies, patients) experiences with these different quality indicators, as well as the latters’ relationship to economic (e.g., cost per patient) and other indicators (e.g., hospital admissions, return to work rates) could solidify our understanding of the positive or negative effects of quality indicators on the practice of occupational therapy and, potentially, other health professions. Also, an in-depth review on the use of structural indicators to evaluate efficiency, appropriateness, and effectiveness of health care service provision and the health care policies they are embedded in would be needed to put evidence on process- and outcome-indicator use into a larger context.

## Conclusion

Among the quality indicators we identified for this report, all have their advantages and disadvantages. The establishment of process-level indicators specific to occupational therapy could be a chance to foster the use of best practice methods, based on available evidence or existing guidelines (see, e.g., [42]).

In terms of outcome indicators, GAS and PRO-Ergo seem to be the most versatile assessments, while also taking into account the Federal Council’s call for patient-centredness in quality assurance [43]. Also, they allow an evaluation on the level of activity and participation (e.g., in work and employment), not solely on the level of body functions and structures (e.g., musculoskeletal functions). As the goals in the GAS are formulated individually, these can include the activity and participation levels as well as body function and structure levels.

While functional outcome indicators (e.g., change in range of motion) present easily understandable data, they are often highly specific to certain client groups and/or health conditions. The definition of functional outcome indicators for every possible client group or health condition may be a disproportionate effort. However, functional outcome indicators that cover a broad area of client problems, like *Section GG* [25] or a similar assessment of ADL, may be useful additional quality indicators for some areas of practice.

There was little information on the use of quality indicators to demonstrate the effectiveness, appropriateness, and efficiency of occupational therapy services to funding bodies in Europe and English-speaking countries abroad that was openly available. This could mean that the use of such quality indicators is either not that widespread, that information on their use is not very accessible, or both. Furthering research in these areas, including patients’ perspectives, and fostering the accessibility of such documents is therefore highly recommended.

The results of this study have practical implications for health care policymakers, professional associations or unions representing health care professionals as well as other stakeholders in the health care field (e.g., insurances). Especially if the goal of quality indicators is maintaining or increasing standards of care, quality indicators should be chosen in order to accurately reflect the specific contributions of different health care professions.

## Data Availability

No datasets were generated or analysed during the current study.

## Abbreviations

ADL: Activities of daily living
CMS: Centers for Medicare & Medicaid Services
DVE: German professional association of occupational therapists
EVS/ASE: Swiss National Association of Occupational Therapy
GAS: Goal Attainment Scale
HIA: Federal Act on Health Insurance (Switzerland)
MACRA: Medicare Access and CHIP Reauthorization Act
MIPS: Merit-Based Payment System
NICE: National Institute for Health and Care Excellence
OAMal: Ordinance on Compulsory Health Care (Switzerland)
OASIS: Outcome and Assessment Information Set
PREM: Patient reported experience measure
QIPOH: Quality Improvement Plan for Ontario Health Teams
QPP: Quality Payment Program
WFOT: World Federation of Occupational Therapists
